# Farm-to-fork investigation of an outbreak of Shiga toxin-producing *Escherichia coli* O157

**DOI:** 10.1099/mgen.0.000160

**Published:** 2018-02-28

**Authors:** Deborah Wilson, Gayle Dolan, Heather Aird, Shirley Sorrell, Timothy J. Dallman, Claire Jenkins, Lucy Robertson, Russell Gorton

**Affiliations:** ^1^​ Public Health England North East Health Protection Team, New castle, UK; ^2^​ Public Health England Field Epidemiology Service, New castle, UK; ^3^​ Public Health England Food, Water and Environmental Microbiology Laboratory, York, UK; ^4^​ Durham District Council Food Safety, Durham, UK; ^5^​ Public Health England Gastrointestinal Bacteria Reference Unit, Colindale, London, UK; ^6^​ Stockton-on-Tees District Council Environmental Health, Stockton-on-Tees, UK

**Keywords:** Shiga toxin-producing *Escherichia coli*, outbreak, contaminated meat products, farm-to-fork

## Abstract

Fifteen cases of Shiga toxin-producing *Escherichia coli* (STEC) O157 infection were associated with the consumption of contaminated food from two related butchers’ premises in the north-east of England. Ten cases were admitted to hospital and seven cases developed haemolytic uraemic syndrome. A case control study found a statistically significant association with the purchase of raw and/or ready-to-eat (RTE) food supplied by the implicated butchers’ shops. Isolates of STEC O157 were detected in two raw lamb burgers taken from one of the butchers’ premises. Subsequent environmental sampling identified STEC O157 in bovine faecal samples on the farm supplying cattle to the implicated butchers for slaughter. Whole genome sequencing (WGS) was performed on the Illumina HiSeq 2500 platform on all cultures isolated from humans, food and cattle during the investigation. Quality trimmed Illumina reads were mapped to the STEC O157 reference genome Sakai using bwa-mem, and single nucleotide polymorphisms (SNPs) were identified using gatk2. Analysis of the core genome SNP positions (>90 % consensus, minimum depth 10×, mapping quality (MQ)≥30) revealed that all isolates from humans, food and cattle differed by two SNPs. WGS analysis provided forensic-level microbiological evidence to support the epidemiological links between the farm, the butchers’ premises and the clinical cases. Cross-contamination from raw meat to RTE foods at the butchers’ premises was the most plausible transmission route. The evidence presented here highlights the importance of taking measures to mitigate the risks of cross-contamination in this setting.

## Data Summary

Short read FASTQ sequences have been deposited in the NCBI Short Read Archive under BioProject PRJNA248042 (http://www.ncbi.nlm.nih.gov/bioproject/248042). Accession numbers are listed in Table S1 (available in the online version of this article).

## Outcome

During an investigation of an outbreak of Shiga toxin-producing *Escherichia coli* O157 associated with the consumption of meat products from two butchers’ premises, whole genome sequencing analysis provided forensic-level microbiological evidence to support the epidemiological links between the clinical cases, the butchers’ premises and the farm supplying cattle to the implicated butchers for slaughter. The most plausible transmission route was cross-contamination from raw beef to other raw and ready-to-eat meat products at the butchers’ premises. The evidence presented here highlights the importance of taking measures to mitigate the risks of cross-contamination in this setting.

## Introduction

Shiga toxin-producing *Escherichia coli* (STEC) O157 cause infections with a broad range of clinical presentations, including bloody diarrhoea and haemolytic uraemic syndrome (HUS). HUS is associated with long-term renal, cardiac and neurological problems and can be fatal, particularly in infants, young children and the elderly [1]. Strains of STEC O157 are highly infectious and fewer than 100 bacteria can cause symptoms. There is a high risk of person- to-person transmission within households and in other settings, such as day nurseries, primary schools, nursing homes and hospitals [1].

Cattle and sheep are the most important reservoir of STEC O157 in the UK, although STEC have also been found in the faeces of a wide range of animals, including deer, rabbits, pigs and wild birds [2, 3]. It is not unexpected or unusual to detect STEC O157 in farm animals or in the farm environment [4]. Infection occurs following consumption of contaminated ready-to-eat (RTE) food or drink, contact with raw meat, faeces from infected animals or people, or contact with an environment contaminated with faeces by an infected animal or person. The incubation period for STEC O157 is usually 3–4 days, but has been recorded as between 1 and 14 days [1].

In July 2015, the Public Health England (PHE) Health Protection Team (HPT) in the north-east of England detected an increase in the number of cases of STEC O157 infection above what would be expected (11 cases within 5 days, compared with a seasonally adjusted expected rate of three per week). Preliminary epidemiological investigations identified the consumption of contaminated meat products purchased from two related local butchers’ premises as a potential source of the outbreak. The aim of this investigation was to describe the subsequent trace-back investigations to identify the origins of the contaminated meat products and to examine the epidemiological and microbiological evidence for the proposed route of transmission from farm-to-fork.

## Methods

### Case ascertainment by enhanced epidemiological surveillance

Presumptive isolates of STEC were reported directly to PHE Centres by clinical microbiologists at local hospital laboratories and a standardized STEC Enhanced Surveillance Questionnaire was administered to cases by environmental health practitioners (EHPs).

### Case control study

Cases were defined as any primary, laboratory-confirmed case of STEC O157, or an individual with HUS, resident in the north-east of England with onset of symptoms between 28 June and 19 July 2015. Controls were recruited from four local general practitioners and frequency-matched to cases by area of residence and age group (aiming for a ratio of three controls per case). A questionnaire was designed capturing information about any diarrhoeal illness and exposures occurring between 28 June 2015 (10 days before the date of onset in the earliest known case) and 16 July 2015 (the date on which control measures were implemented). Exposures included (i) purchase or consumption of raw or RTE food items from a list of butchers in the local area during the exposure period, and (ii) purchase or consumption of specific food items from the implicated butchers (where exposure to these premises was identified), either in the home, institutional setting such as a school or at an event outside the home. Questionnaires were administered by telephone between 16 July and 7 August 2015.

Data were entered into EpiData and analysed using stata 13.1. Analyses included descriptive analysis (distribution of cases and controls by age, sex and postcode of residence) and univariate analyses for association between illness and the four exposures listed above. Exact logistic regression was used to calculate odds ratios (ORs) as the measure of effect, with associated 95 % confidence intervals and *P*-values. Where effect sizes were inestimable due to zero counts, median unbiased estimates (MUEs) were obtained. Adjusted odds ratios (AORs) for age and sex were calculated using binomial regression and associated *P*-values were determined using likelihood ratio testing. Stepwise multivariable logistic regression was also attempted, including variables with OR greater than 1 on a univariate and statistical association of *P*<0.2, and in addition age and sex as possible confounders. However, this was not successful because of the relatively small study population.

### Microbiology of clinical isolates

Isolates of STEC O157 were sent to the Gastrointestinal Bacteria Reference Unit (GBRU) at PHE for confirmation, phage typing (PT) and whole genome sequencing (WGS) [5, 6]. For WGS, DNA was extracted from cultures of STEC O157 for sequencing on the Illumina HiSeq 2500 platform. Quality trimmed Illumina reads were mapped to the STEC O157 reference genome Sakai (GenBank accession BA000007) using bwa-mem [7]. Single nucleotide polymorphisms (SNPs) were identified using gatk2 [8] in unified genotyper mode. Core genome positions that had a high-quality SNP [>90 % consensus, minimum depth 10×, mapping quality (MQ)≥30] in at least one isolate were extracted. SNP positions that were present in at least 80 % of isolates were used to derive maximum-likelihood phylogenies with RaxML [9] using the GTRCAT model with 1000 iterations. FASTQ sequences for all STEC O157 isolates sequenced at PHE have been deposited in the National Center for Biotechnology Information Short Read Archive under bioproject PRJNA248042. The short read accessions of all isolates linked to this outbreak (*n*=22) and those most closely related to the outbreak isolates (*n*=29) are listed in Table S1.

For cases where a faecal sample had not been obtained or was negative for STEC O157, a blood sample was requested and tested at GBRU for serum antibodies to the LPS of *E. coli* O157.

### Food safety and food and environmental samples

EHPs visited the two local branches of the implicated butchers from 15 July onwards to take food samples and swabs of the environment, to observe practice and provide advice on food hygiene. On 22 July, a Veterinary Investigation Officer (VIO) visited the small slaughterhouse at one of the butchers’ premises to observe a slaughter, and EHPs took environmental samples at the slaughterhouse. EHPs took RTE food and raw meat samples, and environmental swabs on multiple occasions including six environmental samples taken from the slaughterhouse and cutting room. Samples were transported to PHE Food, Water and Environmental Microbiology Laboratories at York for microbiological examination in accordance with the Food Standards Agency Food Law Code of Practice (https://www.food.gov.uk/enforcement/codes-of-practice/food-law-code-of-practice-2015).

### Farm sampling

On 23 and 25 September 2015 a VIO from the Animal and Plant Health Agency (APHA) visited the two farms that had supplied cattle to the implicated butchers for slaughter on either 24 June or 1 July. Fifty-two floor and faecal samples taken at Farm 1 and 25 samples at Farm 2 were collected and examined at the APHA Microbiology Laboratories at Bury St. Edmunds, as described previously [10].

## Results

### Descriptive epidemiology

**Fig. 1.**
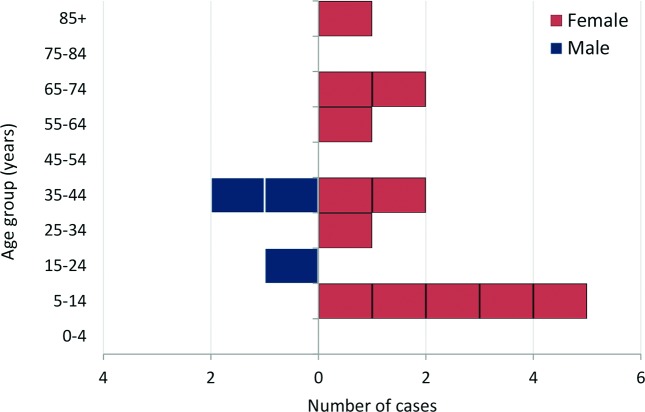
Age and sex distribution of the cases (*n*=15).

**Fig. 2.**
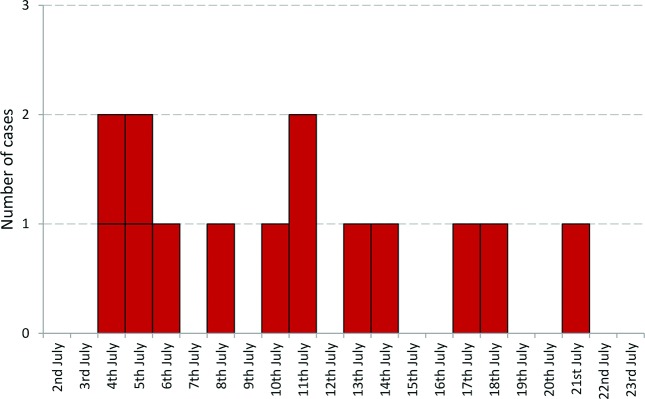
Epidemic curve using onset date (*n*=14).

Fifteen cases were identified, of which 13 were laboratory-confirmed as STEC O157 PT21/28. There were two probable cases; one case did not submit either a faecal or a serum specimen, and the other was diagnosed serologically and had serum antibodies to the LPS of *E. coli* O157. Cases were aged between 6 and 89 years with a median age of 38 years. Three cases (20 %) were male and 12 (80 %) were female (Fig. 1). One of the 15 cases was an asymptomatic household contact of another case. The dates of onset of symptoms for the other 14 cases are shown in the epidemic curve in Fig. 2. The likely incubation period ranged from less than 24 h up to 9 days.

Symptoms reported included diarrhoea (*n*=14), bloody diarrhoea (*n*=13), abdominal pain (*n*=13), nausea (*n*=9), fever (*n*=9) and vomiting (*n*=7). Of the 15 cases, four were not assessed in hospital and one was assessed in hospital but not admitted. Ten cases (67 %) were admitted to hospital and seven (47 %) developed HUS.

Cases consumed a variety of products. One ate only food purchased raw while six cases consumed both raw and RTE foods, and eight cases consumed RTE foods only. No common raw foods were consumed; a range of RTE foods were consumed with cooked sliced ham being the most frequent, consumed by 9/15 cases. All cases apart from one ate food purchased directly from the implicated butchers or through schools supplied by the implicated butchers. The remaining case purchased food from another butcher who had been supplied with meat slaughtered at the implicated butchers; this was the only other butcher so supplied in the outbreak period.

### Case control study

A total of 10 cases (all the known cases at the time the study commenced) and 30 controls were included in the case control study. The demographic characteristics of cases and controls were similar with no significant difference in age. Controls were not matched to cases by gender, enabling the association with female sex to be estimated (OR=7.9). The higher proportion of female cases (90 %) when compared with controls (53 %) was not statistically significant (Fisher's exact test, *P*=0.06). ORs for other associations were adjusted for both age and sex in order to provide reassurance that these were not confounding the observed associations.

All 10 cases (100 %) and eight controls (27 %) reported purchase by a member of their household of raw or cooked/RTE foods from a local butcher or meat retailer within the specified exposure period. Purchases were reported from eight butchers. There was a statistically significant association with purchasing food from the implicated butchers (*P*<0.001), the MUE odds of which were 18 times higher for cases when compared with controls, and adjusted for age and sex (Table 1). The adjusted MUE odds of consuming raw food from the implicated butchers were 45 times higher for cases when compared with controls, and the adjusted odds of consuming cooked food from the implicated butchers were 208 times higher (Table 1). Attempts to look at the association between illness and purchase or consumption of specific food items from the implicated butchers were not successful because of the small numbers of exposed cases and controls.

**Table 1.** Association with sex and consumption of any food, raw or cooked/RTE food by butcher

Exposure		**Cases**			**Controls**			**OR**	**95 %** CI	***P***	AOR*	**95 %** CI	***P***
		**Total**	**Exposed**	**%**	**Total**	**Exposed**	**%**						
Sex	Male	10	1	10	30	14	47	REF			–	–	–
	Female	10	9	90	30	16	53	7.9	0.9–369.3	0.06	–	–	–
Any food	Any other butcher	10	3	30	30	6	20	1.7	0.34–8.68	0.52	1.15	0.2–6.6	0.87
	Implicated butcher	10	10	100	30	2	7	119.9†	14.8–.	<0.001	17.7†	5.0–.	<0.001
Raw food	Any other butcher	10	3	30	30	6	20	1.71	0.34–8.68	0.52	1.15	0.2–6.6	0.87
	Implicated butcher	10	6	60	30	1	3	43.5	4.1–461.2	<0.001	44.6†	5.2–.	<0.001
Cooked food	Any other butcher	10	1	10	30	1	3	3.22	0.18–56.9	0.43	4.16	0.13–134.9	0.41
	Implicated butcher	10	9	90	30	1	3	261	14.8–4607.5	<0.001	208.2	11.5–3780.4	<0.001

*Adjusted for age and sex.

†Median unbiased estimate. The upper limit is inestimable.

Taking exposure to foods from the implicated butchers to be associated with illness, the incubation period for cases included in the case control study (calculated using the latest described date of consumption) ranged from 1 to 5 days (mean 3.5 days, sd 1.6) for raw foods (*n*=6) and 1–7 days (mean 3 days, sd 1.9) for cooked foods (*n*=9) and was in keeping with the estimated incubation period for diarrhoeal illness caused by infection with STEC O157.

### Microbiology

**Fig. 3.**
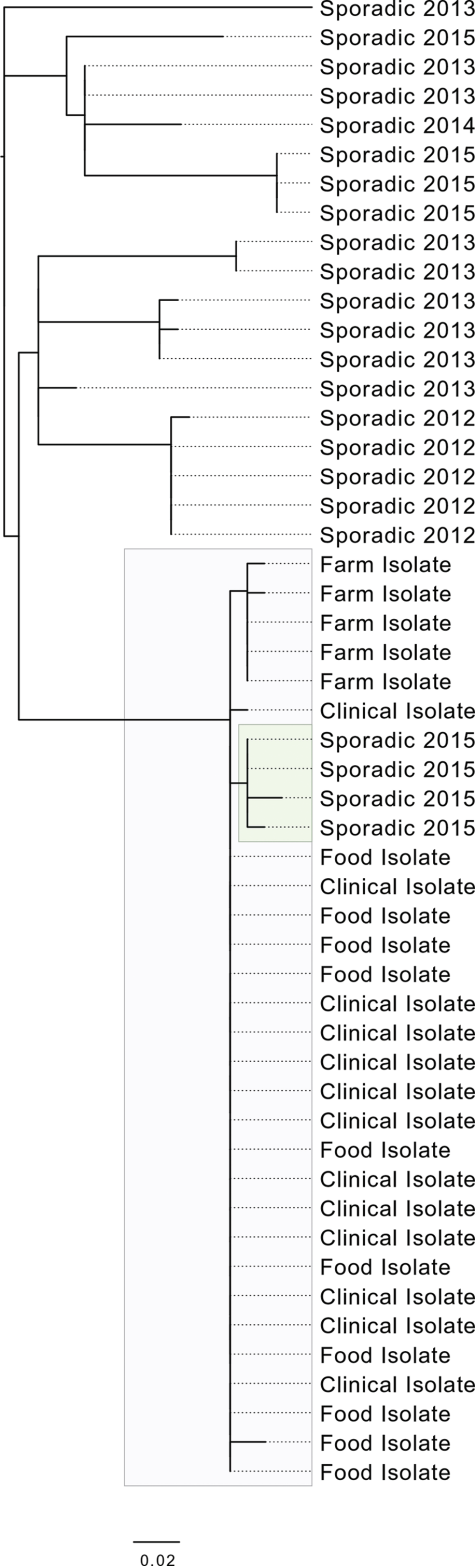
Phylogeny of the clinical, food and farm isolates linked to the outbreak (highlighted in grey) including the four isolates from cases related temporally and geographically to the outbreak but who did not report any links to the butchers’ premises (highlighted in green). Sequences from other closely related sporadic isolates (sporadic isolates being defined here as not linked to the outbreak) within the 25 SNP single linkage cluster available in the GBRU archive are shown for context.

WGS revealed that 12/13 isolates from cases known to be linked to the outbreak were identical at the core genome SNP level and 1/13 was one SNP different from this genotype (Table S1 and highlighted grey in Fig. 3). Isolates from four cases identified through routine surveillance differed from the common outbreak genotype by one shared SNP (Table S1 and highlighted green in Fig. 3). These cases were excluded from the outbreak based on the case definition, as they did not report purchase or consumption of meat products from the implicated butchers’ premises. All four cases were resident in the north-east of England, and reported onset of symptoms between 28 June and 19 July 2015, the same geographical region and time frame as the cases known to be associated with the outbreak. None of the other 18 isolates within the same 25 SNP single linkage cluster as the outbreak strain (Table S1 and Fig. 3) were geographically and temporally related to the outbreak.

### Food safety

The VIO reported being content with the cleaning, measures taken to avoid contaminating meat, use of protective clothing and how the animal by-products were handled at the butchers’ slaughterhouse.

EHPs identified a number of practices that may have facilitated cross-contamination from raw meat to RTE foods at one of the butcher’s premises, including (i) inconsistencies in dilution and contact time of the disinfectant, (ii) inconsistent cleaning of equipment, (iii) use of cloths for both raw and RTE areas/equipment, (iv) excessively hot water for hand washing which could deter effective hand hygiene, (v) evidence of poor handwashing techniques and (vi) equipment used for manipulating raw meat subsequently used for manipulating RTE foods. At the other premises excessively hot water for hand washing was also identified, which may have deterred effective hand washing. EHPs provided advice to reduce these risks and monitored implementation of the advice.

### Food and environmental samples

A total of 142 food and environmental samples were taken as part of this investigation. Colonies of STEC O157 were isolated from two frozen raw lamb burgers made in one of the premises on 8 July and sampled on 20 and 28 July. The burgers were made at the premises using raw lamb minced using the same mincer as used to mince beef, and put into the raw meat display or frozen for sale at a later date. Ten colonies of STEC O157 (five from each of the two lamb burger samples) were found to be PT21/28. Nine of the 10 burger isolates were identical at the core genome level and shared the same SNP genotype with 12/13 isolates from the clinical cases, while one of the 10 burger isolates differed by two unique SNPs from this genotype (Fig. 3). STEC O157 was not isolated from any other food and environmental samples, including those from the slaughterhouse.

### Farm samples

STEC O157 were isolated from 11/52 samples taken from Farm 1. Five of 11 isolates were PT8, one isolate was not typeable and 5/11 were STEC O157 PT21/28. WGS revealed that all farm isolates shared a single SNP different from the common SNP genotype found in the human and food cases (Fig. 3). Trace-back investigations showed that the meat products linked to the outbreak were made from cattle slaughtered 4 months prior to when the cattle on the farm were sampled in September. This time difference may explain the phylogenetic placement of the farm isolates compared to the human and food isolates.

## Discussion

This outbreak was a public health emergency causing serious illness and requiring prompt investigation and control measures. The severity of illness in cases, including 47 % of cases developing HUS, was higher than would be expected for this cohort. All cases consumed food from the implicated butchers, either directly purchased or through a school outlet, or in one case purchased food from the sole butcher supplied with meat from a slaughterhouse at the implicated butchers.

The case control study provided epidemiological evidence to support the conclusion of a link to the implicated butchers’ premises with the odds of consuming any food being 18 times higher for cases when compared with controls and cooked/RTE food being 208 times higher. The predominance of female cases may reflect chance, or differences in patterns of purchase, preparation or consumption of food. Only one case ate only foods purchased raw. There was no common RTE food item consumed by cases, indicating probable contamination of a range of RTE foods. It was not possible to look at the statistical association between illness and purchase or consumption of specific food items from the implicated butchers due to the small study population.

Outbreaks of STEC O157 associated with contamination at a butcher’s premises have been described previously [11, 12]. However, during this investigation, the source of the contaminated food was traced back to the farm supplying the cattle to the implicated butchers for slaughter. WGS data provided microbiological evidence in support of the epidemiological links between the farm, the butchers’ premises and the clinical cases. All isolates from clinical cases, raw lamb burger samples and a farm supplying cattle to the butchers for slaughter clustered together phylogenetically with limited diversity between their genomes (0–2 SNPs). The mutation rate of STEC O157:H7 has been estimated to be in the order of 2.5 SNPs per genome per year [13]. This level of diversity was consistent with the farm-to-fork transmission route exposed by the epidemiological investigation. The finding of the outbreak organism in lamb burgers despite the probable bovine origin indicated by both the farm results and the case associated with consumption of calf liver purchased raw provides further evidence for transmission of the organism between foods within the implicated butchers.

The four clinical cases that clustered with the outbreak cases (highlighted in green in Fig. 3) were likely to have been exposed to STEC O157 from the same source via a different transmission route, for example direct contact with the cattle or their environment, or person-to-person transmission from someone who had consumed contaminated meat from the implicated butchers’ shops. Alternatively, they may have failed to recall, or were unaware, that they had consumed meat from the implicated butchers’ premises.

In England and Wales, the prevalence of excretion of STEC O157 by individual cattle is estimated to be approximately 4.2 %, with approximately 38.7 % of cattle farms having at least one positive animal [14]. In Scotland, the overall prevalence of STEC O157-positive farms was estimated to be 22 % [15, 16]. Samples taken from an abattoir isolated STEC O157 from 1.4 % of 1500 beef carcasses and 0.7 % of 1500 lamb carcasses [17]. In 2003, STEC O157 was identified from 3.2 % of bovine carcasses at a commercial Irish abattoir [18]. Cattle hides have been reported as having higher levels of STEC O157 contamination than carcasses, and figures from studies from the Republic of Ireland and England range from 7.3 to 33 % [19, 20].

Previous studies have found between 0.3 % (3/1015) and 1.1 % (36/3216) of raw beef products sampled in the UK to be contaminated with STEC O157, and 2.9 % (29/1020) of raw lamb products [2, 17, 21]. Raw meat, including offal, can become contaminated with bacteria, including STEC O157, during slaughter or during the processing of carcasses. Control measures to reduce the risk of harm include (i) only accepting clean cattle for slaughter, (ii) using food safety management systems based on Hazard Analysis and Critical Control Points (HAACP) at slaughter, cutting and boning, distribution, retail and catering levels, (iii) maintaining chilled storage, (iv) avoiding cross-contamination between raw and RTE foods and (v) fully cooking minced beef products to a core temperature that will kill any bacteria present. The Food Standards Agency has produced guidance for food businesses on controlling cross-contamination with STEC O157 (https://www.food.gov.uk/sites/default/files/ecoli-cross-contamination-guidance.pdf).

It is not unexpected or unusual to detect strains of STEC O157 on farms and farm animals in the UK, and there is no reliable way to eliminate the presence of STEC O157 in cattle. It is therefore accepted that cattle destined for the food chain may have STEC O157 in their gut or on their hide before leaving a farm or they may become contaminated with the bacteria during transport or lairage before entering the abattoir. Raw meat is not expected to be a sterile product and should always be handled in a manner such that any bacteria present are not spread to RTE foods. During this outbreak, cross-contamination from raw meat to RTE foods at the butchers' premises was the most plausible transmission route, highlighting the importance of measures to prevent any opportunities for cross-contamination between raw and RTE food.

## Data bibliography

1. Dallman, T. J., Ashton, P. A., Jenkins, C., Grant K. NCBI Short Read Archive PRJNA248042 (2015).

## Supplementary Data

43 Supplementary File 1
